# Metastatic Medulloblastoma in Childhood: Chang's Classification Revisited

**DOI:** 10.1155/2012/245385

**Published:** 2011-09-11

**Authors:** Christelle Dufour, Annick Beaugrand, Barry Pizer, Julie Micheli, Marie-Stephanie Aubelle, Aurelie Fourcade, Dominique Couanet, Agnes Laplanche, Chantal Kalifa, Jacques Grill

**Affiliations:** ^1^Department of Pediatric and Adolescent Oncology, Institute Gustave Roussy, 114 rue Edouard Vaillant, 94805 Villejuif, France; ^2^Department of Oncology, Alder Hey Children's NHS Foundation Trust, Liverpool, UK; ^3^Department of Pediatric Hematology/Oncology, CHU Amiens, Amiens, France; ^4^Biostatistics and Epidemiology Unit, Institute Gustave Roussy, Villejuif, France; ^5^Department of Radiology, Institute Gustave Roussy, Villejuif, France

## Abstract

*Purpose*. To correlate the radiological aspects of metastases, the response to chemotherapy, and patient outcome in disseminated childhood medulloblastoma. *Patients and Methods*. This population-based study concerned 117 newly diagnosed children with disseminated medulloblastoma treated at the Institute Gustave Roussy between 1988 and 2008. Metastatic disease was assessed using the Chang staging system, their form (positive cerebrospinal fluid (CSF), nodular or laminar), and their extension (positive cerebrospinal fluid, local, extensive). All patients received preirradiation chemotherapy. *Results*. The overall survival did not differ according to Chang M-stage. The 5-year overall survival was 59% in patients with nodular metastases compared to 35% in those with laminar metastases. The 5-year overall survival was 76% in patients without disease at the end of pre-irradiation chemotherapy compared to 34% in those without a complete response (*P* = 0.0008). *Conclusions*. Radiological characteristics of metastases correlated with survival in patients with medulloblastoma. Complete response to sandwich chemotherapy was a strong predictor of survival.

## 1. Introduction

Medulloblastoma (MB) is the most common malignant brain tumor of childhood. It has a propensity for leptomeningeal spread via the cerebrospinal fluid (CSF) circulation, and approximately 30–35% of the patients present with evidence of metastatic disease [1]. 

Currently, patients with disseminated medulloblastoma are classified according to Chang's operative staging system [2], where the extent of metastasis is subdivided into M0 (no metastasis), M1 (presence of tumor cells in the CSF), M2 (nodular seeding in the cerebellar or cerebral subarachnoid space or in the third or lateral ventricle), M3 (metastasis in spinal subarachnoid space), and M4 (metastases outside the cerebrospinal axis). This sensitive operative system was initially determined from operative records and autopsy specimens and has been later adapted to modern imaging techniques such as MRI. Neither the extent nor the various aspects of the metastases are taken into account, and only the location is considered. The relevance of intracranial (M2) and spinal (M3) leptomeningeal spread for classification as high-risk disease is unequivocal and the outcome for M2 patients is not significantly different from M3 patients [1, 3]. Since the original classification in 1969 that did not separate the outcome of patients with M0 and M1 disease, there is still some uncertainty about the prognostic impact of M1 stage [3–8]. In addition, the assessment of response to therapy of the metastatic disease is often difficult and limited by the sensitivity of MRI [9]. Following personal observations, we made the hypothesis that metastases could be further subclassified on the basis of specific phenotypic criteria with potential correlation with patient outcome. In the present study, we investigated whether the MRI appearances of metastases and the response to chemotherapy could predict survival in disseminated medulloblastoma.

## 2. Methods

### 2.1. Patients

The medical records and imaging of all children with newly diagnosed disseminated medulloblastoma treated at our institution from 1988 to 2008 were reviewed. Inclusion criteria for this study were (1) children <18 years at diagnosis and (2) complete medical records including pre- and postoperative cerebral imaging, analysis of CSF, and interpretable cranial and spinal MRI scans. Exclusion criteria were (1) recurrent medulloblastoma and (2) previous treatment for malignancy. 

Parents/guardians gave written informed consent for the retrospective analysis of clinical data according to the IRB of the Gustave Roussy Institute. For patients entered into ongoing protocols, written informed consent was obtained from their parents/guardians.

### 2.2. Treatment

Children were treated with different protocols. All patients received the same sandwich chemotherapy, with a combination of carboplatin and etoposide [10]. 

The treatment varied with the time and was stratified by age. Patients older than 5 years of age were treated by conventional chemotherapy with craniospinal irradiation (CSI) (30–35 gray (Gy)) [3, 11] or sequential HDCT with ASCT followed by standard dose CSI (36 Gy) [12, 13]. Children younger than 5 years of age were treated by high-dose chemotherapy (HDCT) with autologous stem cell transplantation (ASCT) followed by local radiation therapy to the posterior fossa (50–55 Gy) [12] or sequential HDCT with ASCT followed by reduced dose CSI [14].

### 2.3. Classification Definition and Followup Evaluations

**Figure 1:**
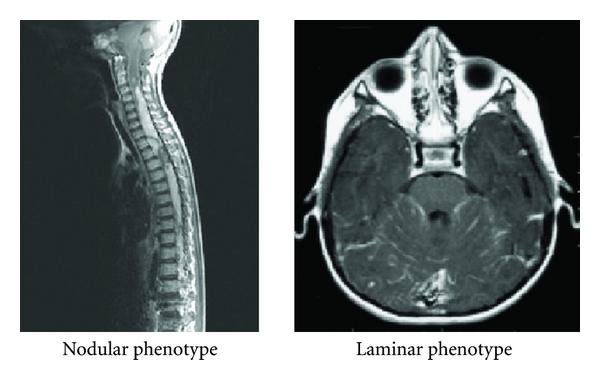
Metastasis phenotype.

Cytological analysis of CSF collected by lumbar puncture was performed between the 7th and 15th postoperative day. The presence of metastases was evaluated by the initial cranial and spinal MRI according to Chang's staging system [2]. Metastases were described according to their phenotype (nodular, laminar) (Figure 1) and their extension (localized, extensive). Nodular metastases were defined as an abnormal deposit with gadolinium enhancement measurable in two dimensions. Laminar metastases were defined as abnormal appearances with gadolinium enhancement that were not measurable in two dimensions. Metastases were classified as laminar where there was coexistence of metastases of laminar and nodular appearance. Extent of disease was defined as “localized” in the case of a single metastasis or in the same area (e.g., periventricular, spinal conus) and “extensive” when there was more than one metastasis in 2 different sites. According to these radiological criteria, we considered two classifications: *qualitative* classification (positive CSF, nodular, laminar) and *quantitative* classification (positive CSF, localized, extensive). 

After each cycle of chemotherapy, cranial and spinal MRI were performed, and children underwent cytological analysis of CSF if initially positive for tumor cells.

### 2.4. End-Points and Statistical Analysis

The final analysis was performed in April 2009. Patients were considered as having incomplete followup if they were not seen 6 months before the time of analysis. 

The potential association of each classification (Chang's, *qualitative* and *quantitative* classifications) with overall survival and with early complete response was investigated as well as the influence of age at diagnosis (age <5 years or age ≥5 years) and treatment. 

Overall Survival (OS) was defined as the time from the date of diagnosis to the date of death or last contact and Progression-Free Survival (PFS) from the date of diagnosis to the date of first recurrence or death.

Early response to chemotherapy was evaluated after the whole preirradiation chemotherapy. Complete response was defined as the total disappearance of a residual primary tumor and metastases (radiological and/or CSF metastases) during or at the end of chemotherapy. Because classical criteria of partial response (> or <50%) are difficult to apply to both leptomeningeal lesions and CSF involvement, data were classified as partial response when radiological response of lesions was observed in combination with a negative CSF cytology, as stable disease in the case of (a) positive CSF at diagnosis (M1) which remained positive or (b) stable radiological imaging (without progression in CSF cytology), and as progressive disease when progression on imaging was noted or when negative CSF cytology became positive. 

Results are expressed as percentages or medians (range). Response rates were compared using nonparametric tests: chi-square or exact Fisher test. OS and PFS were estimated using the Kaplan Meier method [15] and Rothman's 95% Confidence Intervals [95% CI] [16] and compared using the logrank test. Median followup was estimated using the inverse Kaplan-Meier method [17]. In a multivariate analysis of OS, the Hazard Ratios (HRs) of death and their 95% CI were estimated using the Cox proportional hazards model [18]. In a multivariate analysis of complete response, the Hazard Ratios (HRs) of event and their 95% CI were estimated using logistic regression. All reported *P* values are twosided. *P* values below 5% were considered significant. Analyses were performed with SAS version 9.1.

## 3. Results

### 3.1. Patients

117 children with disseminated medulloblastoma were eligible for this study. The median age at diagnosis was 4 years (range, 0–14), and 68 (58%) were less than 5 years. There were 82 males (70%) and 35 females (30%). Thirty-eight children (32%) were treated by conventional chemotherapy (etoposide and carboplatin) followed by CSI at “conventional” doses. HDCT with ASCT followed by local radiation therapy to the posterior fossa was performed in 10 patients (9%). Forty-seven (40%) children received sequential HDCT with ASCT followed by reduced dose CSI, and 22 patients (19%) were treated with sequential HDCT with ASCT followed by standard dose CSI. 

Table 1 presents the pattern of metastatic disease according to the 3 classification systems. Among the 36 patients with nodular metastasis, 15 (42%) were Chang stage M2 stage and 21 (58%) were M3 stage. For the 51 patients with laminar metastasis, 10 (17%) were M2 and 49 (83%) were M3. Of the 26 children with localized metastasis, 18 (69%) were M2 stage and 8 (31%) were M3, and of the 69 with extensive metastasis, 7 (10%) were M2 stage and 62 (90%) were M3. Sixty-one percent of patients older than 5 years of age had nodular metastases.

### 3.2. Progression-Free and Overall Survival

The median followup was 8 years (range, 1–17). For 19 children, the followup was incomplete with a median time between the last followup and the time of analysis of 22 months (range, 7–78 months). Sixty-six deaths were noted: 64 patients from disease and 2 from treatment-related toxicity. Relapse or death as first event occurred in 72 children. There were two cases of second tumor, one with brainstem glioma 58 months after the initial diagnosis and one renal carcinoma 106 months after the initial diagnosis. The estimated five-year overall survival (OS) and progression-free survival (PFS) rates among the 117 children were 45% (95% CI: 36–55%) and 38% (CI 95%: 29–47%), respectively.

### 3.3. Response to Sandwich Chemotherapy

**Figure 2:**
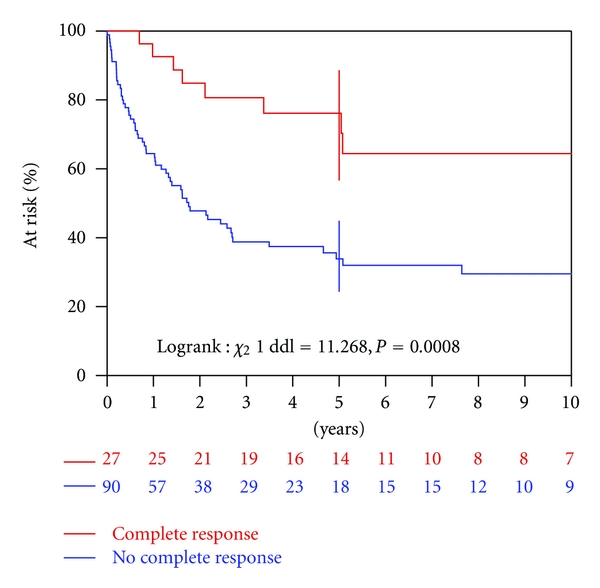
Overall survival according to the early response to sandwich chemotherapy.

The early response rate to sandwich chemotherapy is given in Table 2. The median delay from the date of diagnosis to the date of assessment of response to sandwich chemotherapy was 67 days (range, 26–340). A complete response to sandwich chemotherapy was observed in 27 (23%) of the 117 patients. OS was higher among these 27 patients than among the 90 patients with residual disease, *P* = 0.0008 (Figure 2). The corresponding 5-year OS rates were 76% and 34%, respectively.

### 3.4. Univariate and Multivariate Analysis

Univariate analysis identified age less than 5 years as significantly associated with poor survival and poor early response rate. Overall survival was lower for children younger than 5 years than for those aged 5 or more (HR: 2.5 (1.5–4.2), *P* = 0.001). The HR of no early complete response for children younger than 5 years compared to children of 5 years or more was 3.1 (1.3–7.5), *P* = 0.014. There was no significant association between initial treatment and OS (*P* = 0.290). Thus the multivariate analysis was adjusted for age (<5 years/≥5 years) only.

**Figure 3:**
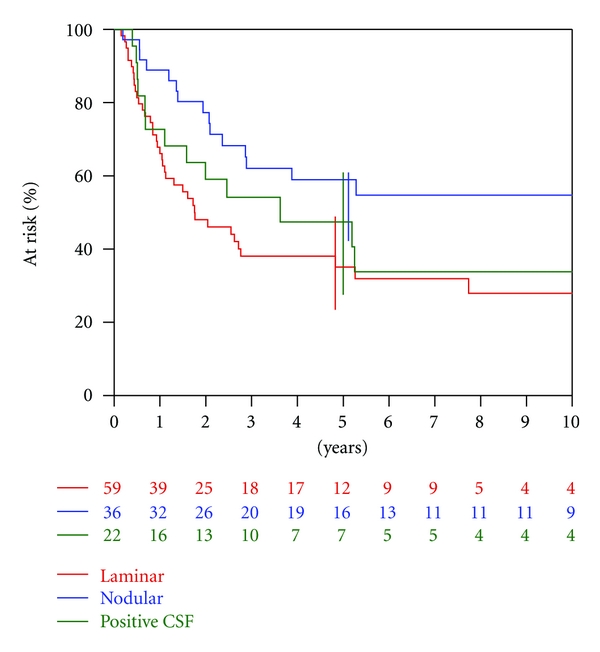
Overall survival according to qualitative classification.

**Figure 4:**
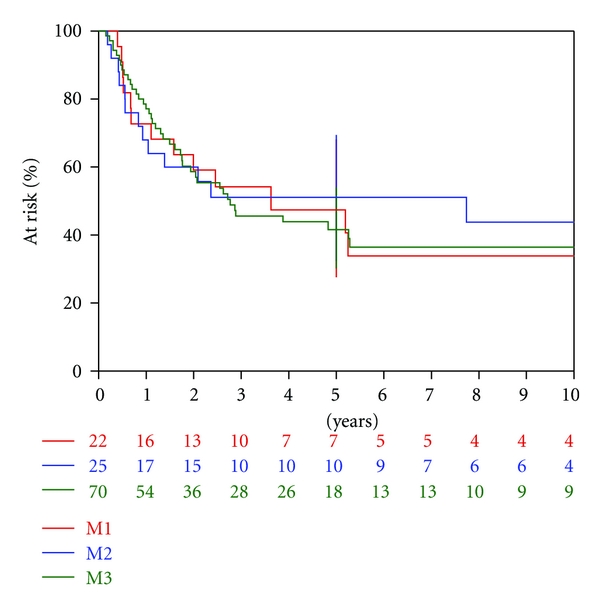
Overall survival according to Chang's classification.

In the univariate analysis, the *qualitative* classification was significantly correlated (*P* = 0.04) with OS (Table 3). OS was higher for children with nodular metastasis (HR = 0.6 (0.3–1.2)) and lower for children with laminar metastasis (HR = 1.3 (0.7–2.5)), compared to children with positive CSF. The five-year OS rates were 47%, 59%, and 35% for patients with positive CSF, nodular metastases, and laminar metastases, respectively (Figure 3). There was no significant correlation of OS with either the Chang classification (*P* = 0.95) or the *quantitative* classification (*P* = 0.12). According to Chang's staging system, the five-year OS rates were 47%, 51%, and 42% for M1, M2, and M3 stage, respectively (Figure 4). 

In the multivariate analyses, after adjustment on age, none of the three classifications was associated with OS, even when *qualitative* and *quantitative* classifications were combined. 

Chang's, *qualitative* and *quantitative* classifications are significantly associated with early complete response rate after sandwich chemotherapy in univariate and multivariate analyses (Table 2).

## 4. Discussion

In this retrospective review of 117 children with disseminated medulloblastoma treated at a single institution, we found that the phenotype of metastasis had an impact on OS. Patient with nodular metastasis had a better survival than the patients with other metastatic groups. Diffuse metastases (i.e., laminar metastases, M1 disease) were associated with a more aggressive disease than those with nodular disease. The impact of the phenotype of metastases on OS has not been published previously. In this study, the *qualitative* classification was significantly correlated with OS (*P* = 0.04) in univariate but not in multivariate analysis. Since the phenotype of the metastases was associated with age in our study, we may have lost its effect on prognosis by adjusting the multivariate analysis on age. Moreover, since response to chemotherapy is associated with the phenotype of the metastases and is the strongest prognostic factor identified in our study, different treatment policies according to age may confound the results of the multivariate analysis. We can hypothesize that there are intrinsic biological differences linked to age that drive the phenotype of the metastases and their response to chemotherapy. These results should be confirmed in a large prospective study. 

The Chang's operative staging system was initially shown to predict outcome according to the T stage, that is, tumor size and invasion, although recent publications have failed to show an impact of T stage on survival [19, 20]. The Chang system did not, however, include prognostic information with respect to the different M stages. Subsequent studies have clearly shown the adverse prognostic factor of the presence of metastasis. In many studies, the high-risk group was defined as Chang M2 and M3 stage, and the outcome for M2 patients was not significantly different from M3 patients [1, 3, 8, 21, 22]. There remained doubt, however, about the prognostic significance of M1 stage and whether patients with M1 disease constituted a truly high-risk group. Several studies have found an intermediate risk for M1 metastases, with outcomes rates lower than in M0 but superior to M2/3 dissemination [3–8]. In our study, we found that patients with M1 disease (with/without residual mass) are truly high-risk patients with no significantly different outcome compared with solid metastases. Sanders et al. showed recently that patients with M1 disease have reduced rates of EFS and OS compared to those with M0 disease [7]. Children younger than 3 years of age with M1 MB had significantly decreased EFS compared to those with M0 disease and in fact fared as poorly as those with macroscopic metastatic disease (M2/M3) [7]. On the other hand, in the German HIT 91 trial, patients with M1 disease had a similar outcome to those with M0 with 3-year EFS of 72%, 65%, and 30% for patients with M0, M1, and M2/3 disease, respectively [23]. 

One has to take into account the type of treatment since the HIT'91 trial randomizing sandwich against maintenance chemotherapy found a better outcome for the M1 patients in the maintenance arm (i.e., with early radiotherapy) and a better outcome for the M2/M3 patients in the preirradiation chemotherapy arm. Since our policy was to give the same sandwich chemotherapy to all patients, this may have been detrimental to M1 patients, while beneficial for M2/M3 patients. 

The earlier response to chemotherapy seems to be important for outcome. We found that patients in complete remission after preirradiation had significantly a better OS compared those not in complete remission. The prognostic impact of early response to chemotherapy found in our study is consistent with others' reports [24, 25]. 

In summary, findings in this study suggest that the phenotype of metastases should be taken into account when describing a population of children with metastatic medulloblastomas. If confirmed by further prospective studies, this report suggests that treatment strategies for metastatic medulloblastoma need to be refined taking into account the nature as well as the presence of metastases.

The early response to sandwich chemotherapy may also help us to stratify the treatment of children with disseminated medulloblastoma. Further studies are justified to find biological correlates with respect to the metastatic phenotype.

## Figures and Tables

**Table 1:** *Qualitative* and *quantitative* classifications according to Chang's.

		*N* (%)		
Chang's classification		M1 22 (19%)	M2 25 (21%)	M3 70 (60%)

*Qualitative* classification				
Positive CSF:	22 (19%)	22 (100%)		
Nodular:	36 (31%)		15 (42%)	21 (58%)
Laminar:	59 (50%)		10 (17%)	49 (83%)

*Quantitative* classification				
Positive CSF:	22 (19%)	22 (100%)		
Localized:	26 (22%)		18 (69%)	8 (31%)
Extensive:	69 (59%)		7 (10%)	62 (90%)

**Table 2:** Univariate and multivariate analysis of early Complete Response (CR) to sandwich chemotherapy.

		Univariate analysis			Multivariate analysis		
	CR (%)	HR	IC (95%)	*P* value	HR	IC (95%)	*P* value
Chang'staging							
M1 (*n* = 22)	11 (50%)	1		*0.006*	1		*0.002*
M2 (*n* = 25)	3 (12%)	7.3	1.7–31.8		9.8	2.0–46.9	
M3 (*n* = 70)	13 (19%)	4.4	1.6–12.3		6.7	2.1–21.6	

*Qualitative* classification							
Positive CSF (*n* = 22)	11 (50%)	1		*0.005*	1		*0.002*
Nodular (*n* = 36)	8 (22%)	3.5	1.1–11.0		6.3	1.7–23.7	
Laminar (*n* = 59)	8 (14%)	6.4	2.1–19.5		8.1	2.4–27.2	

*Quantitative* classification							
Positive CSF (*n* = 22)	11 (50%)	1		*0.003*	1		*0.001*
Localized (*n* = 26)	7 (27%)	2.7	0.8–9.0		4.2	1.1–15.8	
Extensive (*n* = 69)	9 (13%)	6.7	2.2–19.8		9.7	2.9–32.9	

**Table 3:** Univariate and multivariate analysis of Overall Survival (OS).

		Univariate analysis			Multivariate analysis		
	5-year OS	HR	IC (95%)	*P* value	HR	IC (95%)	*P* value
Chang's classification							
M1 (*n* = 22)	47%	1		*0.95*	1		*0.73*
M2 (*n* = 25)	51%	0.9	0.4–1.9		0.9	0.4–2.0	
M3 (*n* = 70)	42%	1.0	0.5–1.9		1.2	0.6–2.2	

*Qualitative* classification							
Positive CSF (*n* = 22)	47%	1		*0.04*	1		*0.17*
Nodular (*n* = 36)	59%	0.6	0.3–1.2		0.7	0.3–1.6	
Laminar (*n* = 59)	35%	1.3	0.7–2.5		1.3	0.7–2.4	

*Quantitative* classification							
Positive CSF (*n* = 22)	47%	1		*0.12*	1		*0.16*
Localized (*n* = 26)	65%	0.6	0.3–1.3		0.7	0.3–1.5	
Extensive (*n* = 69)	36%	1.2	0.6–2.2		1.3	0.7–2.4	
